# Impact of TNF and IL-33 Cytokines on Mast Cells in Neuroinflammation

**DOI:** 10.3390/ijms25063248

**Published:** 2024-03-13

**Authors:** Pio Conti, Gianpaolo Ronconi, Dorina Lauritano, Filiberto Mastrangelo, Alessandro Caraffa, Carla E. Gallenga, Ilias Frydas, Spyridon K. Kritas, Francesco Carinci, Federico Gaudelli, Ciro Annicchiarico, Cristian D’Ovidio

**Affiliations:** 1 Immunology Division, Postgraduate Medical School, University of Chieti, 66100 Chieti, Italy; 2 Clinica dei Pazienti del Territorio, Fondazione Policlinico Gemelli, 00185 Rome, Italy; gianpaolo.ronconi@policlinicogemelli.it; 3 Department of Translational Medicine, University of Ferrara, 44121 Ferrara, Italy; dorina.lauritano@gmail.com; 4 School of Dentistry, University of Foggia, 71100 Foggia, Italy; filibertomastrangelo@hotmail.com (F.M.); federico_gaudelli.563780@unifg.it (F.G.); 5 School of Pharmacy, University of Camerino, 62032 Camerino, Italy; alecaraffa@libero.it; 6 Section of Ophthalmology, Department of Biomedical Sciences and Specialist Surgery, University of Ferrara, 44121 Ferrara, Italy; gllcln@unife.it; 7 Department of Parasitology, Aristotle University, 54124 Thessaloniki, Greece; stavfd1991@gmail.com; 8 Department of Microbiology and Infectious Diseases, School of Veterinary Medicine, Aristotle University, 54124 Thessaloniki, Greece; skritas@vet.auth.gr; 9 Department of Morphology, Surgery and Experimental Medicine, University of Ferrara, 44121 Ferrara, Italy; crc@unife.it; 10 Independent Researcher, 70100 Bari, Italy; annicchiarico.ciro63@gmail.com; 11 Section of Legal Medicine, Department of Medicine and Aging Sciences, G. D’Annunzio University of Chieti-Pescara, 66100 Chieti, Italy; cridov@yahoo.its

**Keywords:** inflammation, cytokines, TNF, IL-33, mast cells, microglia, brain disorders

## Abstract

Mast cells (MCs) are derived from hematopoietic progenitors, mature in vascularized tissues, and participate in innate and acquired immunity. Neuroinflammation is a highly debated topic in the biomedical literature; however, the impact of tumor necrosis factor (TNF) and IL-33 on MCs in the brain has not been widely addressed. MCs can be activated by IgE binding to FcεRI, as well as by different antigens. After activation, MCs mediate various immunological and inflammatory responses through TNF and IL-33. TNF has two receptors: TNFR1, a p55 molecule, and TNFR2, a p75 molecule. This cytokine is the only one of its kind to be stored in the granules of MCs and can also be generated by de novo synthesis via mRNA. In the central nervous system (CNS), TNF is produced almost exclusively by microglial cells, neurons, astrocytes, and, minimally, by endothelial cells. After its release into brain tissue, TNF rapidly induces the adhesion molecules endothelial leukocyte adhesion molecule 1 (ELAM-1), intercellular adhesion molecule 1 (ICAM-1), and vascular cell adhesion molecule 1 (VCAM-1) in endothelial cells. TNF causes the chemoattraction of neutrophils by inducing several molecules, including CXC chemokines (IL-8). Both MCs and microglial cells act as a primary barrier against foreign molecules in the CNS, producing pro-inflammatory cytokines such as IL-33. IL-33 belongs to the IL-1 family, is activated through the ST2L/IL1-RAcP receptor complex, and mediates both the innate and adaptive immune response. IL-33 is a nuclear transcription factor expressed in the brain, where it induces pro-inflammatory cytokines (TNF and IL-1) and chemokines (CCL2, CCL3, CCL5, and CXCL10). Therefore, MCs and microglia in the CNS are a source of pro-inflammatory cytokines, including TNF and IL-33, that mediate many brain diseases. The inhibition of TNF and IL-33 may represent a new therapeutic approach that could complement existing neuroinflammatory therapies.

## 1. Introduction

Focal inflammation of the brain is a pathology that is difficult to understand, and the treatments available today are not very effective [1]. Neurons control the entire human organism through a complex and still largely unclear mechanism [2]. Tissue inflammation and inflammation in many brain diseases is the body’s defensive reaction that takes place in an attempt to oppose damage to the central nervous system (CNS) [3].

Inflammation is a risk factor implicated in neurological diseases such as Alzheimer’s Disease (AD), Parkinson’s Disease (PD), Multiple Sclerosis (MS), dementia, and stroke, in addition to many others. In this paper, we focus on the pathogenesis of brain inflammation, leaving aside the study of the individual disease at a neurological–inflammatory level. Genetic and environmental factors are implicated in the development of neurological diseases, and the pathogenesis is still unclear. Several published papers have reported that mast cells (MCs) are involved in brain inflammation. MCs are hematopoietic-derived immune cells present at the perivascular level in various areas of the brain and in the meninges, where they express a number of molecules when activated, including the cytokines tumor necrosis factor (TNF) and IL-33.

Neurological disorders present neuroinflammation mediated by MCs. For example, MCs were found in greater numbers in CNS lesions in patients suffering from MS and were found to produce higher levels of tryptase [4]. Experimental autoimmune encephalomyelitis (EAE) is an experimental model that mimics MS in some ways. Animals affected by chronic EAE show neuropathological dysfunction of the retina and nerves. It has recently been reported that MCs may play an important role in EAE, although this hypothesis is controversial. Mouse models with MC deficiency have allowed scientists to study the role of these cells in MS and EAE in more depth. Recent studies have suggested that MCs can not only have an inflammatory function but also a protective immune function. However, mouse models have been very useful for investigating the role of MCs in MS and EAE and for improving the pathogenetic knowledge of the disease, although many questions still need to be clarified.

Neuroinflammation involves different immune cells, such as microglia, T cells, neutrophilic granulocytes, and MCs [5]. MCs store granular cytoplasmic TNF, which can be rapidly secreted after activation, and they are also responsible for a later synthetic release that occurs hours after cellular stimulation. TNF activates MCs to generate other pro-inflammatory cytokines [6].

MC progenitors, which respond to stem cell factor (SCF), are bone marrow-derived cells that migrate in vascularized tissue, where they undergo the maturation process [7]. They are found close to the blood vessels, gastrointestinal tract, epithelial nerves, brain, and other sites, where they act as immune sentinels [8]. Upon IgE FcεRI activation, MCs generate a spectrum of pro- and anti-inflammatory molecules that participate in different diseases [9] (Table 1). In addition to allergic diseases, MCs play an important role in neurological disorders such as AD, PD, and MS, amongst others. This activation causes the aggregation of receptors, which results in the quick release of preformed mediators stored in MC granules. In addition, they can be activated by many antigens, including microorganisms which bind Toll-like receptor (TLR)4 and cause the generation of pro-inflammatory cytokines and chemokines [10].

MC activation occurs after physical, chemical, or biological damage. It is a defense process of the body that causes the release of various molecules and chemical mediators, including proteases, vasoactive amines, proteoglycans, proteolytic enzymes, prostaglandin D2 (PGD2), and the leukotrienes LTC4, D4, and E4, which are all mediators of inflammation [11]. In the inflammatory network, the chemokines RANTES and MCP-1 are released and attract MCs, basophils, neutrophils, monocytes, and lymphocytes [12].

**Figure 1:**
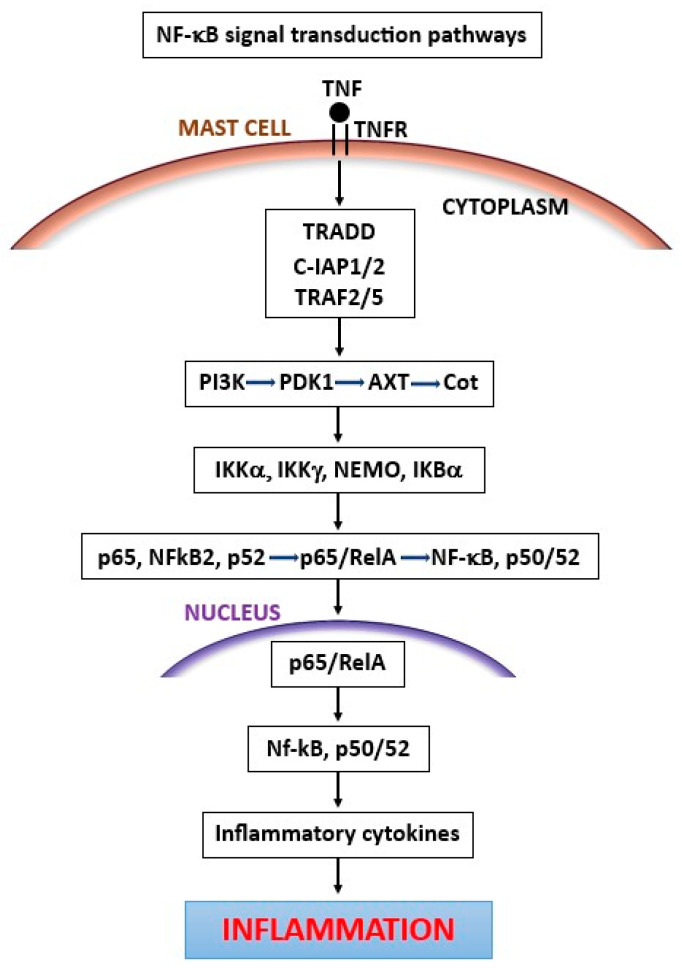
TNF binds to its receptor and activates NF-κB signal transduction pathways, leading to the release of pro-inflammatory cytokines.

Macrophages collaborate with MCs in inflammatory processes, and they are great producers of cytokines, which can also activate them [13]. In fact, bacteria, or bacterial products [such as lipopolysaccharide (LPS)], activate the CD14 macrophage receptor by binding TLR 2 or 4, leading to the production of cytokines and reactive oxygen species generation, along with the consequent killing of the microbe [14]. Phagocytes express several receptors that can recognize microbes such as TLRs, G protein-coupled receptors, Fc receptors, C3 receptors, and cytokine and interferon-gamma (IFN-γ) receptors [15]. The TLRs expressed by both macrophages and MCs belong to a family of approximately 10 proteins and are sequentially named from 1 to 10 (for example: TLR1, TLR2, TLR3, etc.) [16]. Cells of the innate immune system, such as macrophages, neutrophils, dendritic cells (DCs), epithelial cells, endothelial cells, and MCs, all express TLRs, molecules that are similar to the IL-1 and IL-18 receptors [17]. The TLRs of human cells respond to antigens expressed in microbes and stimulate the inflammatory response to microorganisms [18]. TLR acts through the activation of nuclear factor kappa B (NF-κB) or the mitogen-activated protein kinase (MAPK) cascade, which leads to the activation of the transcription factor activation protein-1 (AP-1) [19]. The TLR response leads to the coding of inflammatory cytokine genes for TNF, IL-1, and IL-18, as well as for adhesion molecules such as E-selectins and proteins that are involved in the defense against microbes [20]. TNF secreted by MCs may have an autocrine effect which, through binding to the TNF receptor of MCs, can activate NF-κB signal transduction pathways, leading to the secretion of inflammatory cytokines [21] (Figure 1).

## 2. Mast Cell Activity

**Figure 2:**
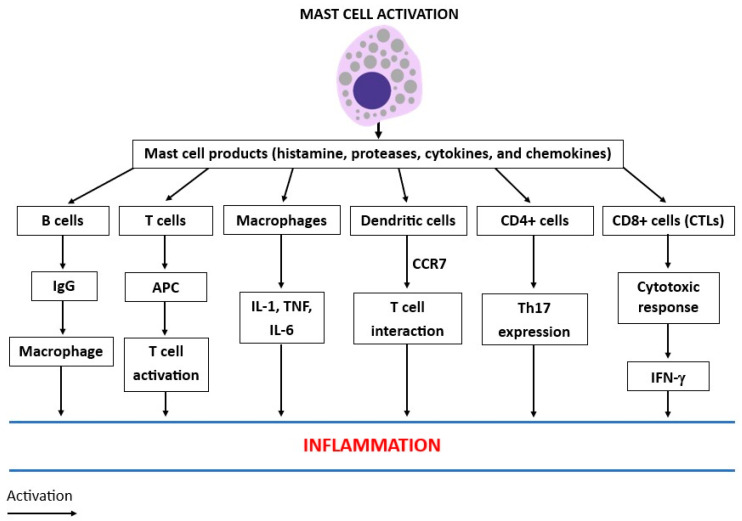
Activated mast cells (MCs) release diverse compounds, including histamine, proteases, cytokines, and chemokines, which activate different immune cells that participate in inflammatory reactions.

MCs participate in both innate and acquired immunity. IgE is a classic activator of MCs, and it mediates immunological responses after binding to their FcεRI receptors, promoting acute tissue swelling, local fibrin deposition, and inflammation [22]. Human MCs express c-kit, activated by the c-kit ligand, which is the most important growth factor receptor [23]. The suppression of c-kit by tyrosine kinase inhibitors causes the apoptosis of MCs and reduces inflammation in several disorders [24]. In addition, MCs can be activated by antigen-specific mediator release, such as the IgG1 TH2 response, by using the FcγRIII receptor [25]. Therefore, IgE and IgG1 antibodies participate in the induction of the inflammatory reaction. IgE and the antigen-dependent activation of MCs may also contribute to cell-mediated immune functions [26]. MCs have hematopoietic origins and produce biologically active molecules such as stored mediators, PGD2, LTs, cytokines, and chemokines [27]. In addition, they generate caspase-1, which participates in the maturation of IL-1β, a key cytokine in inflammation, pain, and fever [28]. Therefore, MCs are the source of other cytokines that participate in immune regulation and have potential pro- and anti-inflammatory activity [29]. They can be activated by different stimuli that act on different pathways [30]. MCs can crosstalk with other cell types such as DCs, macrophages, B cells, CD4+ cells, and CD8+ cells. CD8+ cells express many subsets of both TH1- and TH2-type cells, which generate pro-inflammatory cytokines (such as IFN-γ) and produce cytokines (such as IL-9, IL-17A, and IL-22), respectively. All the white immune cells cooperate in the activation and release of several biological compounds, including cytokines/chemokines, which recruit specific immune cells to the site of inflammation (Figure 2).

In inflamed allergic tissue, MCs reside near infiltrating T cells, which release several cytokines, including IL-3, and some chemokines, such as MIP-1α, MCP-1, RANTES, and growth factors [12]. All these cytokines, chemokines, and growth factors contribute to the activation and proliferation of MCs. MCs communicate with activated T cells through the release of inflammatory molecules such as histamine, TNF, and matrix metalloproteinase-9 (MMP-9), which are involved in matrix remodeling and leukocyte trafficking in inflammation [31]. MC products can influence T lymphocytes in the inflammatory response and may cause antigen presentation via MHC I or MHC II, promoting T cell migration through cytokine generation [32]. T cells induce MC activation to produce IL-4 and MMP-9 and facilitate TNF generation, which mediates inflammation [33]. MCs can also release the leukotriene LTB4, which causes cell aggregation and chemotaxis [34]. The products released from MCs can upregulate the expression of adhesion molecules such as the selectins intercellular adhesion molecule 1 (ICAM-1) and vascular cell adhesion molecule 1 (VCAM-1) in endothelial cells [35]. Brain MC activation by neurotransmitters such as substance P evokes an immunoinflammatory response that results in leukocyte adhesion to the venular endothelium and the degranulation of MCs [36].

## 3. Tumor Necrosis Factor (TNF)

**Figure 3:**
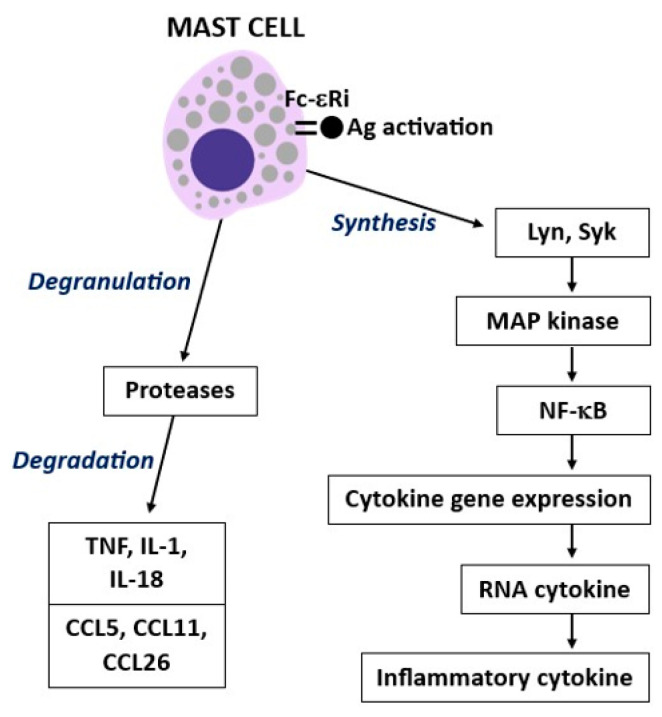
Antigen Fc–εRI receptor activation causes protein degranulation, which degrades inflammatory cytokines and chemokines. Additionally, MC activation leads to NF-κB cytokine gene expression, along with the consequential release of inflammatory cytokines.

Cytokines perform a protective action for the body, but when they exceed physiological levels, they are highly inflammatory and can even lead to death. TNF is a cytokine released by macrophagic cells, lymphocytes, and MCs after activation, and it is generated in cytoplasmatic granules [37]. TNF is a 26 kDa membrane-bound protein with releasing steps that are regulated via the protein kinase C (PKC) α-dependent pathway, and p38 regulates the transport of mRNA from the nucleus to the cytosol [38]. TNF is the only cytokine stored in the granules of human MCs [39]. The granules of antigen-activated MCs promptly release TNF in seconds or through mRNA after hours [40]. The MC-activated gene expression of RNA cytokines leads to the generation of pro-inflammatory cytokines (Figure 3). TNF contributes to the immunological and inflammatory responses, and it is involved in cachexia, strong cell decay, as well as cytotoxicity. Like some other cytokines (IL-1, IL-6, and IFN-γ), TNF has a wide range of actions and is produced by the CNS and peripheral tissues with pleiotropic functions, influencing multiple phenotypic traits [41]. Almost all white immune cells, including MCs, produce TNF; while TNF is produced in the brain almost exclusively by microglial cells, neurons, astrocytes, and, minimally, by the endothelial cells of the cerebral microcirculation [42]. TNF interacts with two receptors, TNFR1 and TNFR2, mediating acute and chronic inflammation, apoptosis, and necrosis, and it has a homeostatic and immune function against pathogenic microorganisms [43]. The TNF rapidly released by MCs initiates innate and adaptive immune responses against microorganisms and causes inflammation [44]. Neurons, endothelial cells, and immune cells primarily express TNFR2, whereas TNFR1 is expressed in all cell types [45]. MCs can rapidly secrete granular cytoplasmic TNF and are responsible for a later synthetic release that occurs after stimulation [46]. TNF activates MCs to generate other pro-inflammatory cytokines; this process allows for the recruitment of neutrophilic granulocytes that initiate inflammation [47]. On the other hand, under certain circumstances, the TNF that is expressed by MCs binding to its receptor in an autocrine manner may support TH2 cell production by promoting pathway remodeling and may ameliorate the inflammatory allergic reaction caused by IgE binding to the FcεRI receptor [48].

**Figure 4:**
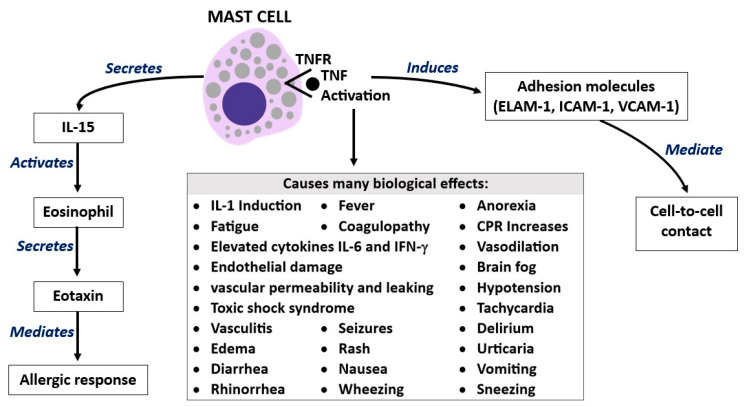
The activation of mast cells (MCs) by TNF leads to the secretion of IL-15, which activates eosinophil to secrete eotaxin, which mediates the allergic response. Moreover, this MC activation induces the adhesion molecules ELAM-1, ICAM-1, and VCAM-1, which mediate cell-to-cell contact. TNF-MC receptor binding causes many biological effects, as reported here in this figure.

TNF released from MC granules in the brain and other tissues rapidly induces the adhesion molecules endothelial leukocyte adhesion molecule 1 (ELAM-1), ICAM-1, and VCAM-1 in endothelial cells [49]. Preformed TNF is contained in the granules of MCs and can be rapidly released after appropriate activation, while de novo TNF protein synthesis requires several hours [29]. Moreover, MC activation secretes the chemokine eotaxin and the cytokine IL-15, which induce eosinophil migration and activation that participate in late allergic responses [50] (Figure 4).

At low concentrations, TNF is important as an immune cytokine against foreign agents, transformed cells, and cancer cells, while at high concentrations, it is highly inflammatory and can even be lethal by mediating toxic shock syndrome [51]. TNF can be constitutively stored in the granules of some MCs, where it can be triggered by substance P, which is produced by certain neurons [52]. MCs exert bidirectional interactions with certain nerve cells, which is an interesting effect for physiological and pathological studies of the CNS [53]. For example, brain MCs participate in crosstalk with neurons and can influence neuronal activity and calcium fluxes; vice versa, neurotransmitters can act on MC receptors and activate them to produce inflammatory molecules. The physiological immune protection of MCs is also exerted by their production of proteases, which can inhibit inflammation by degrading certain pro-inflammatory cytokines, such as TNF, IL-1, and IL-18, and some chemokines, such as CCL5, CCL11, and CCL26 [54].

TNF blockers have been approved as anti-inflammatories for clinical use and also in CNS disorders, and they are particularly used in inflammatory cases where other drugs have given poor results [55]. TNF has an active inflammatory function in neurological diseases including MS and EAE. The biology of anti-TNF in the CNS is very complex, but its therapeutic efficacy in inflammatory diseases is quite well established, even though it can present adverse effects. TNF antagonists represent an important therapeutic option in inflammatory diseases, and in transgenic mice lacking TNF, there is a reduction in the progression of EAE. The treatment of autoimmune neurological diseases such as MS with anti-TNF is gaining traction in clinical practice. However, TNF blockers are still disputed, and more research is needed.

The production of TNF in the brain appears to be related to the protection and survival of neurons that can die in CNS diseases [56]. The cerebral protection exerted by TNF occurs after the activation of the p55 receptor and the secretion of TNF by neurons [57].

MCs produce various growth factors, cytokines, and chemokines and can be potent mediators of inflammation, but they can also perform beneficial immune actions in host defense, both after activation with IgE and against some types of parasites or bacteria [58]. MCs activated by IgE or other molecules generate many chemical mediators and inflammatory proteins, such as cytokines, including TNF. In experimental models on mice, it was reported that MCs produced TNF after activation with bacterial products and the FcεRI receptor [59]. In the inflammatory response, TNF acts on the migration of DCs involved in adaptive immunity and recruits immune cells such as neutrophils, an effect that is inhibited by neutralizing TNF antibodies [60]. The generation of TNF by MCs occurs after antigen or IgE stimulation via affinity receptor FcεRI, which is an important interaction that can lead to acute and chronic inflammation.

There are two TNF receptors: TNFR1, a p55 molecule that is expressed in a wide range of cell types, and TNFR2, a p75 molecule expressed in a limited range of cell types, such as leukocytes and epithelial cells [61]. TNF regulates adhesion molecules and causes the migration of inflammatory cells. It causes the chemoattraction of neutrophils by inducing several molecules, including CXC chemokines, which play an important role in inflammation [62]. IL-8 is a CXC chemokine that induces C-C chemokine activation to recruit monocytes to the inflammatory site [63]. Blocking TNFR1 inhibits the chemoattraction of neutrophils and the activity of IL-8 [64]. Moreover, TNF-derived MCs require the endothelial adhesion molecule E (EAM-E), P-selectin, VCAM-1, and platelet endothelial cell adhesion molecule-1 (PECAM-1) to induce cell-to-cell contact [65]. The PECAM-1 gene is a member of the immunoglobulin superfamily found on the surface of platelets, monocytes, neutrophils, and some types of T-cells [66]. It is involved in leukocyte migration, angiogenesis, and integrin activation [67]. MC-derived TNF regulates the activity of DCs participating in cell-mediated immunity. MCs express TLRs in their surface membranes, which allow them to react against microorganisms as a protective immune response. TLR4 or CD48 activation leads to the secretion of TNF by MCs after antigen binding, an important reaction for immune cell activation and lymphocyte recruitment [68]. Moreover, the secretion of TNF by MCs seems to be crucial for developing and protecting the immune response to bacteria and viruses [69]. Therefore, through the recruitment of DCs, MCs are protective in the innate immune response and also play a role in adaptive immunity.

The release of TNF from MC granules demonstrates that this cytokine plays a key role in the pathogenesis of immune and inflammatory diseases. In addition, brain inflammation, neurotoxicity, and neurodegeneration are mediated by pro-inflammatory cytokines, including TNF [70]. TNF induces endothelial upregulation, leukocyte rolling, and cell adhesion, pro-inflammatory effects that can be reversed by several proteins, including platelet-activating factor (PAF) [71]. Various cytokines participate in the inflammatory network, including TNF, IL-1β, IL-6, and IL-33, as well as the chemokines CCL2 and CCL5 [72]. Metalloproteinases (MPs), granulocyte monocyte colony-stimulating factor (GM-CSF), and glial maturation factor also participate in the inflammatory process, increasing intracellular calcium and activating MAPKs and NF-κB [73]. Together, T lymphocytes, microglia, astrocytes, neurons, and activated MCs secrete inflammatory factors that mediate neurodegeneration and, therefore, brain dysfunction [74]. In addition, the breakdown of the blood–brain barrier (BBB) allows for the passage of inflammatory molecules transported within the circulatory system, which contributes to the pathological state [75]. BBB disruption can be mediated by oxidative stress, metalloproteases, microglial activation, and immune cell infiltration into brain tissue. The mechanisms of BBB disruption are still poorly understood, even though this process has been shown to cause the infiltration of activated immune cells, producing inflammatory molecules, which is the first warning sign for MS and possibly other neurological diseases. This inflammation is often not inhibited by the anti-inflammatory drugs available today, and therefore, this mechanism is still being studied. However, since inflammatory cytokines play an important role in this process, using anti-inflammatory cytokines as a treatment option should be helpful. Brain T lymphocytes that remain in the white matter also produce inflammatory cytokines including TNF and IFN-γ, an effect that causes the activation of inflammatory cells in the CNS [76]. Moreover, specific antigens of the neuronal system can bind to MCs, causing their immediate activation with the production of inflammatory molecules, including the TNF stored in their cytoplasmatic granules [77]. Therefore, in the CNS, MCs and the microcirculation of inflammatory mediators play a role in the pathogenesis of autoimmune diseases such as encephalomyelitis.

MCs are recruited through products generated by T cells which, upon activation, release cytokines, fuel vascular permeability, and upregulate ICAMs [78]. The vascular permeability observed in wild-type mouse models is controlled by endothelial cells and is not only mediated by TNF but also by histamine, bradykinin, and serotonin produced by MCs in the CNS microcirculation [79]. Inflammatory mediators such as histamine, released by the granules of MCs, spill onto the endothelial surface and activate molecules such as P-selectin, a type-1 transmembrane protein that functions as a cell adhesion molecule on the surfaces of activated endothelial cells. In neurological disorders, vascular permeability can be mediated by various immune cells, including neutrophils, which are recruited to the CNS and release inflammatory molecules such as reactive oxygen species (ROS), proteases, and pro-inflammatory cytokines and chemokines [80]. Activated neutrophils create endothelial cell dysfunction and increased permeability with edema formation [81]. T cells participate in the recruitment of neutrophil granulocytes, and it has been noted that CD4+ lymphocytes play an important role in increasing vascular permeability. Damage to endothelial cells leads to abnormal blood flow, increased vascular permeability, probable clot formation, fluid and protein loss, and tissue and organ dysfunction. MC-derived TNF can influence cells of the adaptive immune system, as well as non-immune cells. TNF appears to participate in nerve development through the induction of nerve growth factor (NGF) in keratinocytes, a reaction that has been noted in inflamed skin [82]. The response of MCs via LPS, complement, or TLR4 activation molecules leads to the defensive production of TNF. In an interesting article, it was reported that KitW-sh/W-sh mice engrafted with MCs indicated that MCs-derived TNF can increase mortality during bacterial infection [83]. Proteases produced by MCs can degrade TNF, inhibiting inflammation and increasing survival. However, experiments on genetically modified mice deficient in MCs may not represent the effects seen in humans. Human MCs are genetically different than murine MCs, but even between the different mice type strains, MCs may respond differently. For example, it has been reported that 129/Sv, C57BL/6, and 157 129/Sv mice can produce different levels of histamine [84].

Therefore, inflammatory immune cells and their products are important candidate elements in mediating cerebral vascular pathophysiology.

## 4. IL-33

In the brain, active reactions occur in both innate and acquired immunity. MCs, as well as microglia, are abundant in the meninges, where they act as a primary barrier against foreign molecules and microorganisms and can mediate inflammatory processes. Activated MCs express the FcεRI receptor and produce inflammatory mediators including tryptase, histamine, and serotonin (in mice), but they may also play a protective role [85].

**Figure 5:**
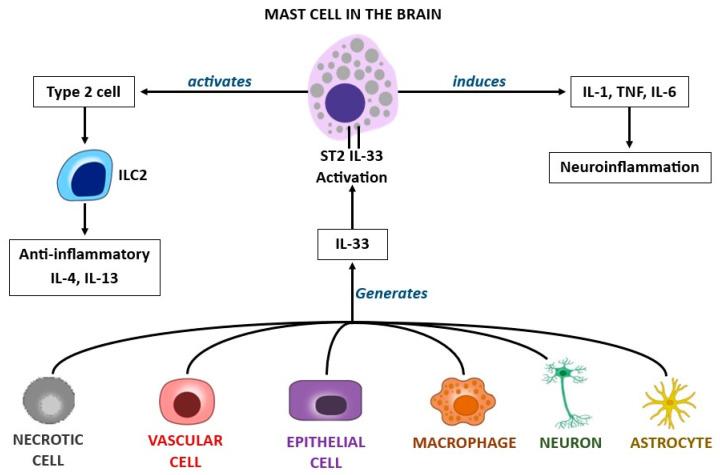
IL-33, which is released by different cells, such as necrotic cells, vascular cells, epithelial cells, macrophages, neurons, and astrocytes, binds to the ST2 receptor. This can produce a dual effect, with the activation of anti-inflammatory cytokines or the secretion of pro-inflammatory cytokines that mediate neuroinflammation.

IL-33 (also called IL-1F11 or NF-HEV) is a cytokine that belongs to the IL-1 family and activates MCs by binding to the ST2L/IL1-RAcP receptor complex by inducing Th2 cytokines [86]. In addition, IL-33, the ligand of the high-affinity receptor ST2, stimulates chemokine production and plays a role in adaptive and innate immunity. IL-33 is a nuclear transcription factor whose full length is confined in the nucleus and consists of up to 270 amino acids which interact with the NF-κB transcription factor p65 subunit [87]. In the CNS of rodents, IL-33 is expressed in the spinal cord and brain, where it induces other pro-inflammatory cytokines and chemokines [88]. Microglia activated by IL-33 induce the cytokines TNF and IL-1 and the chemokines CCL2, CCL3, CCL5, and CXCL10, fueling the inflammatory state [89]. This cytokine has been described as an “alarmin” molecule, and it is generated by MCs secreted by damaged or necrotic cells, and it is also associated with damage-associated molecular patterns (DAMPs). The IL-33 cytokine can also be generated by other activated cells, including vascular, epithelial, and macrophagic cells. Both the ST2 receptor and the protein IL-33 are expressed in the CNS by endothelial cells, neurons, astrocytes, and microglia and are linked to neurological diseases. IL-33 plays a key inflammatory role in hypersensitivity and infectious diseases but also mediates protective immune effects [90,91]. Therefore, it can exert a dual function by acting as a pro- or anti-inflammatory cytokine (Figure 5). T-type immune cells play an important role in the CNS and can be activated by antigens presented by macrophage, dendritic, or glial cells. Although IL-33 is a member of the IL-1 family and mediates innate immune responses, it is mainly involved in Th2-type immune responses. It appears that IL-33 activity is associated with T regulatory (Treg) cells, and so it is possible to find IL-33 in the CNS even in the absence of inflammation [92].

In addition, IL-33 participates in the inflammatory reaction and plays an immune role by activating innate lymphoid cell 2 (ILC2) [93]. ILC2 cells mediate diverse processes, including the generation of IL-4 and IL-13 cytokines, airway remodeling, the stimulation of IgE production, and the activation of T lymphocytes in the Th2 direction [94]. The anti-inflammatory cytokines IL-4 and IL-13 are generated by natural killer (NK) cells, Th2 CD4+ cells, ILC2 cells, basophils, and eosinophils after appropriate activation and are promoted through the phosphorylation of STAT6, which increases the expression of GATA binding protein 3 (GATA3) [95,96]. However, type 2 cytokines can also be expressed without the activation of STAT6 and GATA3, which are transcription factors for the differentiation of Th 2 cells. In addition to anti-inflammatory IL-10, type 2 cytokines are targetable candidates for the amelioration of type 2 inflammatory brain diseases. Almost all neurological diseases present an inflammatory state, some with a higher degree, and others with a lower degree of inflammation. Common neurological disorders include migraine, epilepsy, encephalitis, meningitis, AD, PD, amyotrophic lateral sclerosis, MS, stroke, and dementia, amongst others.

IL-33 plays a fundamental role in the initial stages of type 2 inflammation, not only by acting on ILC2 but also by directly stimulating MCs [97]. In the CNS, IL-33 is highly expressed by glial cells, activates ILC2, and plays an important role in brain diseases. The inflammatory IL-33 produced by MCs participates in the activation of astrocytes with high levels of NF-κB and p38 and the consequent production of inflammatory cytokines [98]. The inhibition of IL-33 can reduce the recruitment of pro-inflammatory cytokines such as IL-1, TNF, and IL-6 with an improvement in neuroinflammation. In addition, IL-33 acts synergistically with IL-25 to produce IL-5 and IL-13 in ILC2 cells [90]. IL-5, which acts through its receptor formed by two IL-5Rα sub-units and a β chain, is the cytokine responsible for the growth and differentiation of eosinophils in the bone marrow, as well as for their mobilization and survival at the bone marrow level of the bloodstream [99]. IL-5 blockade leads to an eosinophilic non-response after an allergenic stimulus [100]. IL-5 is also implicated in IL-3 signaling and macrophage- and granulocyte-stimulating factor signaling.

In addition to being present on the surface of eosinophils and their precursors, the IL-5 receptor is also expressed by basophils [101]. IL-13 is involved in the recruitment of eosinophils from the bloodstream to tissues. This cytokine increases the expression of both VCAM-1, which favors the adhesion of eosinophils to the endothelium, and chemokines such as eotaxin-1 and eotaxin-3, promoting the migration of cells from the vessel to the inflamed tissue [102]. IL-13 can stimulate mucus cells via Notch2 pathways [103]. The Notch2 gene encodes a member of the Notch family which is a type 1 transmembrane protein with an extracellular and an intracellular domain.

The supernatant of activated MCs stimulates TH17 cells to produce the cytokine IL-17 [104]. This is a response that appears to involve IL-1, since blocking the latter cytokine inhibits IL-17 production [105]. This effect implicates MCs as immune cells that are involved in the T cell response. Upon stimulation, TH17 cells, a subpopulation of CD4+ T cells, produce IL-17, a highly inflammatory cytokine with effects on the cells in many tissues. IL-17 mediates the pathogenesis of various immune-mediated and inflammatory diseases, such as rheumatoid arthritis, psoriasis, asthma, and MS [106,107]. The upregulation of neuroinflammation is due to the increased immune response, the activation of microglial cells, the activation of ILC2 and ROS, and the dysregulation of cellular metabolism. It is known that antigen-presenting cells (APCs) crosstalk with the immune responses exerted not only by T and B lymphocytes but also by ILC2 cells and MCs [108]. These immune cells are found in the CNS, where they are abundant in the hippocampus and thalamus, where they produce molecules that mediate brain diseases. The protective and pathological function of MCs is carried out by ILC2 cells. ILC2 cells (ILC1, 2, 3), along with NK cells, are part of a group of innate immune cells that express the CD45 receptor [109]. ILC2 cells express numerous surface receptors, including IL7R, IL2R, IL25R, IL4, IL4R, IL9R, IL10R, and IL33R, and play an important role in inflammatory brain diseases by producing neuroinflammatory cytokine mediators such as IL-33 [110]. In contrast, IL-33 is deficient in some neurological diseases, such as in AD, and treatment with IL-33 can improve the cognitive and pathological symptoms in mouse models [111] (Table 2). In brain diseases, the ST2 receptor is activated by the consequent production of IL-33, promoting the secretion of IL-13, which is associated with type 2 inflammation and is an ideal target for inhibiting the inflammatory response [112].

At the brain level, the activation of ILC2 cells with IL-33 inhibits the production of pro-inflammatory cytokines and enhances the pathophysiological effects [118]. Therefore, the activation of ILC2 cells can improve the inflammatory state in experimental neurological diseases with a nonspecific pleiotropic effect.

## 5. Conclusions

Cerebral inflammation, also known as neuroinflammation, occurs in many neurological pathologies, including AD and MS, and includes reactions that tend to inflict damage on the CNS. The function of immune cells that regulate brain physiology is still unclear. However, in experimental mouse models, ILC2 cells in the aged brain, activated by IL-33, seemed to improve cognitive function [119]. The protective inflammatory reactions are mediated by immune cells including microglia and MCs that generate pro-inflammatory cytokines. Activated MCs generate TNF, IL-33, cytokines, and chemokines that aggravate the inflammatory process, although IL-33 generated by ILC2 cells can also act by inhibiting inflammation. The activation of MCs can also produce other inflammatory molecules, such as members of the IL-1 family, arachidonic acid products such as the prostaglandin PGD2, and the leukotrienes LTC4 and LTD4.

It is well established that MCs are important effector cells mediating inflammation through the production of inflammatory cytokines such as TNF and IL-1. A therapeutic strategy that could help in neurological diseases could be to specifically block these cytokines produced by MCs with other anti-inflammatory cytokines.

In this article, we have reported that neuroinflammation is mediated by TNF and IL-33 that is generated by activated MCs.

## Figures and Tables

**Table 1:** Mast cell mediators.

*Stored in the cytoplasmatic granules:* Histamine, serotonin (in mice), tryptase, chymase, peroxidase, heparin, chondroitin Sulphates, hydrolases, carboxypeptidases.
*Cytokines secreted after appropriate activation:* TNF, IL-1, IL-3, IL-4, IL-5, IL-6, IL-8, IL-10, IL-9, IL-11, IL-12, IL-13, IL-15, IL-16, IL-18, IL-25, TGF-β, VEGF, IFN-γ, GM-CSF, (and probably many more).
*Chemokines:* CCL1, CCL2, CCL3, CCL4, CCL7, CCL8, CCL9, CCL17, CCL20, CCL22, CXCL2, CXCL8, Eotaxin 1 and 3.
*Arachidonic acid mediators after activation:* PGD2, PGE2, LTB4, LTC4

**Table 2:** Some biological effects involving the activity of IL-33 in the brain.

IL-33 regulates the overexpression of pro-inflammatory molecules in the brain [113].
In CNS glia, mast cells (MCs) treated with IL-33 produce TNF and the chemokines CCL17 and CCL11 [114].
In the glial system, IL-33 protein expression is increased by pathogen-associated molecular patterns (PAMPs) [115].
IL-33 induces the secretion of IL-1, TNF, and IL-10 in microglia [92].
In microglia, IL-33 enhances NO synthesis, phagocytic activity, and the synthesis of chemokines such as CCL2, CCL3, CCL5, and CXCL10 [92].
IL-33 regulates the levels of inflammatory molecules by acting on microglial phagocytosis [111].
IL-33 modulates microglia in the production of NLRP3 and reduces the production of the cytokines IL-1 and IL-6 [111].
IL-33 is highly expressed in certain neurological diseases and appears in the plasma of the peripheral circulation [116].
IL-33 and its receptor ST2 are elevated in acute and chronic brain lesions of certain neurological diseases, including multiple sclerosis (MS), where it may inhibit myelination [117].

## Data Availability

Not applicable.
